# Evaluation of the online-based self-help programme “Selfapy” in patients with unipolar depression: study protocol for a randomized, blinded parallel group dismantling study

**DOI:** 10.1186/s13063-021-05218-4

**Published:** 2021-04-09

**Authors:** Rico Krämer, Stephan Köhler

**Affiliations:** Department of Psychiatry and Psychotherapy, Charité – Universitätsmedizin Berlin, Campus Mitte, Charitéplatz 1, 10117 Berlin, Germany

**Keywords:** Major depressive disorders, Internet-based intervention, Blended treatment, Specialized mental healthcare, Routine practice, Randomized controlled trial

## Abstract

**Background:**

Patients with mild to moderate depressive symptoms can have limited access to regular treatment; to ensure appropriate care, low-threshold treatment is needed. Effective online interventions could increase the supply of low-threshold treatment. Further research is needed to evaluate the effectiveness of online interventions. This study aims to evaluate the online-based self-help programme “Selfapy” on a sample of depressive subjects and compares the impact of the programme’s unaccompanied version with its therapeutic accompanied version.

**Methods:**

A sample of 400 subjects that have a mild to severe depressive episode (Becks Depression Inventory - II and Hamilton Depression Scale) will be used. Subjects are randomly assigned to immediate access to an unaccompanied course (no support from psychologist via weekly phone calls), immediate access to an accompanied course (support from a psychologist via weekly phone calls) or a waiting list control group (access to the intervention after 24 weeks). The intervention will last for a period of 12 weeks. Depressive symptoms as a primary parameter, as well as various secondary parameters, such as life satisfaction, therapeutic relationships, social activation, self-esteem, attitudes towards Internet interventions and drop-out rates, are recorded at four different points in time: at baseline (T1), 6 weeks after the start of the intervention (T2), 12 weeks after the start of the intervention (T3) and 3 months after completion of the treatment follow-up (T4).

**Conclusion:**

This randomized and controlled, blinded study will make use of a “dismantled” approach to adequately compare the accompanied and unaccompanied versions of the intervention. Positive and meaningful results are expected that could influence the acceptance and implementation of online interventions.

**Trial registration:**

German Clinical Trials Register DRKS00017191. Registered on 14 June 2019

## Background

Depression is a common and severely distressing condition with a high mortality rate due to suicide and other factors [1, 2]. Depressive disorders are one of the largest global burdens with around 350 million people worldwide, while mild to moderate forms (29% of primary care patients) occur even more frequently than severe depressions (only 9.5% of primary care patients) [3, 4]. Subclinical depression refers to those who do not fully meet the criteria of a depressive episode (ICD-10) or major depression (DSM-5) [5] and is associated with considerable impairment, economic costs and increased risk for developing major depression [4, 6, 7].

Different treatment methods (e.g. cognitive behaviour therapy or pharmacotherapy) were shown to be effective in the treatment of depression [8]. Patients with mild to moderate depressive symptoms do not meet the requirements for currently available options of effective treatment [9, 10]. As there are too few options with low-threshold access, these patients do not receive treatment. There are few randomized controlled trials (RCTs) for low-threshold Internet interventions for those who have mild to moderate depressive symptoms. Low-threshold psychosocial interventions, including online self-help programmes, can be used as a complementary treatment, especially in the case of mild depression [11]. In general, over 20 RCTs, several meta-analyses and systematic reviews in the past have proven their effectiveness in the reduction of depressive symptoms compared to different control conditions (e.g. [12]). Drop-out rates in web-based interventions are typically high, especially in self-guided web-based interventions [13].

Cognitive behaviour therapy (CBT) is ideal for the implementation of online interventions due to its highly structured, directive and standardized nature, as well as its focus on psychoeducation and homework [9]. A systematic review and meta-analysis including 8 randomized studies with regard to Internet-delivered cognitive behaviour therapy (iCBT) was carried out by Zhou et al. [14]. Compared to the inactive control groups, the iCBT programmes were significantly more effective (SMD = − 0.28, CI [− 0.42, − 0.14]; *I*^2^ = 49%) in reducing depressive symptoms.

There are already a few online interventions for depression, but more high-quality trials are needed to prove efficacy [9–11, 15]. Online programmes are offered with or without therapeutic support. Therapeutic support can be delivered with the accompaniment of a clinical psychologist (accompanied) or in combination with conventional face-to-face therapy (blended). Unaccompanied programmes show small effect sizes in the treatment of depressive symptoms compared to the control group [16]. Unlike unaccompanied self-help programmes, accompanied self-help approaches involve regular contact with a clinical psychologist [17]. Accompanied self-help approaches also differ with respect to the intensity of therapeutic contact [15]. Accompanied online depression interventions show medium to large effect sizes compared to the control group [18, 19]. Meta-analyses provide some evidence that programmes that combine elements of traditional face-to-face therapy with an online programme are more effective than unaccompanied online programmes [18, 20]. Further research is necessary to draw conclusions regarding the differences in efficacy and impact between an accompanied and an unaccompanied online self-help programme.

The aim of this study is to validate the effectiveness of the online self-help programme “Selfapy” in an RCT. “Selfapy” offers the possibility of comparing an accompanied and unaccompanied version within the same programme. A randomized, controlled parallel-group study will be performed, and the differences between accompanied and unaccompanied versions of the courses, as well as their effects on treatment outcomes, will be evaluated.

### Trial objectives

With this trial, we evaluate the efficacy of the online self-help programme “Selfapy” and deduced the following hypotheses. The framework of the trial is superiority. The Becks Depression Inventory - II (BDI-II) [21] thereby serves as the primary outcome measure and assesses the depressive symptomatology.

#### Main hypotheses

1. Depressive symptoms (BDI-II scores) will be significantly lower in both study groups following the 3-month “Selfapy” programme compared to the control group.
2. We expect higher depressive symptoms (BDI-II scores) in the unaccompanied group compared to the accompanied group.

#### Secondary hypotheses

1. The drop-out rate is expected to be higher for the unaccompanied group than for the accompanied group.
2. The strength of the therapeutic relationship will be significantly higher in the accompanied group than in the unaccompanied group.
3. Positive attitudes towards Internet interventions will increase for the accompanied group as well as for the unaccompanied group when compared with reported attitudes at baseline. Increased scores in positive attitudes towards Internet interventions will be significantly higher for the accompanied group than for the unaccompanied group.
4. Quality of life, social activation and self-esteem scores will increase significantly over the duration of the 3-month programme compared to scores taken at baseline and compared to scores in the control group. The increase in these parameters will be significantly higher for the accompanied group than for the unaccompanied group.

## Methods

### Recruitment

A total of 400 subjects with mild to severe depressive symptoms are recruited via the “Selfapy” website, advertising on German television and in numerous information brochures from health insurance companies. The central recruiting tool is a study website (www.selfapy.de/studie/), where interested people can register to participate. If a person decides to participate by giving their consent, they will be immediately directed towards the determination of the deadline for the incoming survey (Mini International Neuropsychiatric Interview (MINI) [22], Hamilton Depression Scale (HRSD-24) [23]). Electronic informed consent has been recognized by previous ethics committees as a safe and ethical means of giving consent [24] and is used in this study. Additional consent would be required for the use of patient data in additional or supplementary studies.

### Inclusion and exclusion criteria

To clarify the inclusion and exclusion criteria, interviews will be conducted with all subjects via telephone (MINI, HRSD-24).

All MINI and HRSD-24 interviews will be conducted by the same two trained interviewers, who are a psychologist and a medical student. The training of the interviewers was delivered at the Charité Department of Psychiatry and Psychotherapy. Within this training, an opportunity to practise with depressive patients was provided, followed by a discussion regarding the ratings guided by a psychiatric consultant.

To be eligible, participants must (1) be between 18 and 65 years old, (2) have sufficient German language skills, (3) have uninterrupted access to the Internet, (4) score 12 or higher on the BDI-II, (5) provide their electronic data with the “declaration of consent” and (6) have a major diagnosis of a depressive episode or dysthymia (ICD-10: F32; F33; F34) (according to the MINI).

Subjects were excluded if they met one of the following criteria: (1) diagnosis of a bipolar disorder; (2) currently present psychotic symptoms or a diagnosis of an acute schizophrenic disorder (ICD-10: F2x; F31; F32.3; F33.3), acute substance dependence or withdrawal syndrome (ICD-10:F1x.2; F1x.3); or (3) they are currently experiencing or have experienced suicide ideations (operationalized via HRSD-24—subjects are excluded if they have a score of 3 or above on suicidality items) in the past.

A comorbid diagnosis other than depression is not an exclusion criterion, as the aim was to reflect routine care as realistically as possible. However, an active substance dependency is an exclusion criterion, as psychotherapy is not indicated during an active substance dependency as this limits any intervention (e.g. intoxication, concentration deficit, withdrawal symptoms). In addition, acute addiction could distort the effectiveness of the programme in patients with depression. Other primary psychiatric disorders are excluded as including these patients will reduce the internal and external validity of our trial, which targets the effects of the programme for patients with depressive disorder.

Subjects who do not meet our inclusion criteria due to the severity of illness are encouraged to seek professional help.

Adequate language skills are determined during the initial diagnosis by the MINI and HRSD-24. Our inclusion criteria were critically discussed in the study board.

### Randomization and blinding

Subjects who meet the inclusion criteria will be randomly allocated to one of the three groups: (a) immediate access to the unaccompanied depression course of “Selfapy”, (b) immediate access to the accompanied depression course of “Selfapy” or (c) access to “Selfapy” after a delay of 24 weeks (control group).

**Fig. 1:**
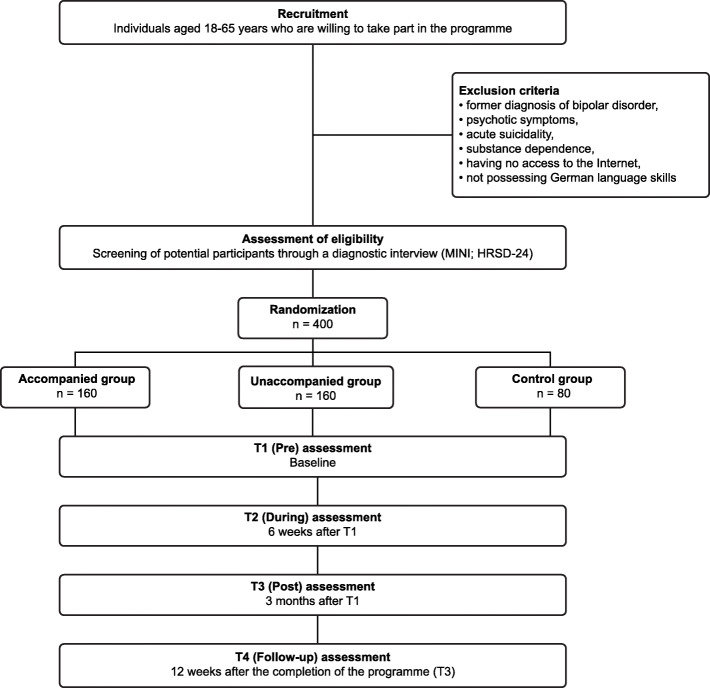
“Selfapy” programme flowchart

Block randomization is performed by an independent researcher using a random number assignment plan for this list, which was created by a computer-controlled random number generator. These numbers are assigned blindly, i.e. subjects and researchers have no knowledge of which group a patient was allocated to. The subjects are randomly assigned to one of the three groups in a ratio of 2:2:1. The subjects will be informed by email about the result of the allocation process. Subjects in one of the intervention groups received an email with a link and their respective access code to register and be able to start the intervention immediately. Subjects in the control group who also received an email with a link that leads the subject to the assessment material will be informed by email about the result of the random assignment. Therefore, participants enrolled themselves in the study programme. Diagnostic interviewers are blind to the assigned group of subjects. An illustration of the patient flow can be found in Fig. 1.

After successfully completing the MINI and HSDR-24, the unblinding of the researchers will be possible in the case of acute suicidality and before the study programme will be terminated for the respective participant.

### Intervention

“Selfapy” is a browser-based commercial programme for reducing depression symptoms (https://www.selfapy.de). To achieve this goal, the user receives instructions on evidence-based methods and exercises in the areas of CBT, system therapy, behaviour therapy and mindfulness training. The “Selfapy” programme is available in an accompanied and unaccompanied version.

Subjects in the intervention groups (accompanied and unaccompanied) have free access to the 12-week Internet-based self-help treatment. The online course is divided into 12 different modules. Every week, a new topic is discussed, such as “self-awareness”, “challenging negative thoughts” or “sociability”. The weekly modules consist of informative texts, videos, interactive exercises and worksheets. Table 1 contains the 12 module topics and a brief description of these and the exercises they contain. The programme also includes a so-called classroom by allowing the psychologist to follow the programme of the subjects. The “classroom” includes an integrated chat system through which psychologists and subjects can communicate.

**Table 1:** Overview of course content

Module	Content	Exercises
Your beginning	A questionnaire is used to collect general data about this person and his/her problems.	Lifeline; problem cakes; miracle question
**Target selection**		
First insights	In this module, the user is brought closer to the connection between thinking, feeling and acting. Based on this triangular connection, our course concept is explained to him/her.	Your personal triangles
Resources	Power sources are explained and gathered. Different methods are introduced to assist users in collecting power sources. Behavioural activation is prepared.	Evaluation daily protocol; power source images; treasure chest; resource walk
Behavioural activation	How to actively plan power sources during the day is explained.	Activity plan
Automatic thoughts	Automatic thoughts and thinking patterns are addressed.	Automatic thoughts; core beliefs
Realistic thoughts	The content of this module focuses on thinking about reality and thinking realistically.	Think realistically
Self-esteem	A positive self-concept is important for overcoming problems. The user learns how the concepts of self-confidence and self-acceptance can help to develop a positive self-image.	Positive self-image
Self-efficacy	The user is made aware of recent successes. This will enable him or her to better allocate his or her power reserves and increase confidence in his or her abilities.	“Your personal achievements—be proud of yourself”
Solve problem	Training to perceive a specific problem, gather potential responses in a problematic situation and develop the competence to implement a particular course of action to solve this problem optimally is introduced.	Problem-solving training
Mindfulness + excursus: pleasure training	In this module, mindfulness is the focus. A mindful approach is cultivated to improve stress management.	Mental note; mindfulness in everyday life; sitting meditation; body scan; your enjoyment moments
Social environment + stigma + social support	The focus of this module is on the environment and the social environment of the user. How social support can help to deal appropriately and successfully with stress is clarified.	“Difficult communication—your backing”
Relapse cases	Finally, a tool is introduced to help the user recognize when stress increases and becomes too much and how to handle it when the stress cannot be avoided.	Risk situations; early warning symptoms; first aid plan; relapse protocol

Subjects are advised to spend at least 15 to 20 min a day on the programme. If the modules are not processed for 3 weeks (recorded via login), this will be counted as the subjects drop out.

### Accompanied group

In the accompanied version of the programme, the subjects are supervised by experienced psychotherapists in training throughout the duration of the programme. In the beginning, the expert and the subject get to know each other and consequently hold 20- to 25-min conversations by phone on a weekly basis. In the course of this conversation, the psychotherapists address the content from Table 1 in a structured manner. A special focus lies on discussing and reviewing the accomplishment of the exercises incorporated in each module*.* In a 1-h training session, all clinical psychologists who were interested in accompanying subjects were informed. The training included general information on the study, risks and handling of these risks, a discussion of the non-accident concept, instructions on the handling and documentation of drop-outs and information on the standardization of the discussion content.

### Unaccompanied group

In the unaccompanied version, the subject works independently throughout the programme.

A chat function with a psychotherapist in training is available in the “classroom”, which can be used to clarify questions for improved understanding. There is no content-based discussion about the chat. The psychologist was also trained here.

### Control group

At the end of the follow-up period, subjects in the control group will receive free access to the online intervention programme. They are free to choose the version of the programme (accompanied or unaccompanied) they prefer. The control group received isolated, nonspecific mood-stabilizing activities such as body scan instructions, guided abdominal breathing and guided imagery. Subjects in the control group will not receive treatment or support from the researchers. However, they are free to seek any other help including pharmacological and psychological treatments. All concurrently used treatments will be measured repeatedly through self-report. Subjects in the control group will have access to the intervention after the last follow-up assessment, 12 weeks after the baseline assessment. The control group could not participate in “Selfapy” within 24 weeks. Duplicate registrations are prevented by comparing email addresses. Moreover, in the interviews, subjects are asked whether they registered more than once.

### Responses to crises and suicidality

A physical overuse can be excluded. The online survey at the time of measurement T1, T3 and T4 of the survey will take about 60 min (Table 2). The processing time for T2 is about 30 min. A general side effect of the interventions could possibly be a lack of treatment success. The most severe adverse event that could happen is acute suicidality. Suicidality is monitored using the BDI-II (after 6, 12 and 24 weeks) and the HRSD-24 (after 12 and 24 weeks). In case of a score of 3 (suicidal thoughts or behaviour) on item 3 on the HRSD-24 or in case of a score of 2 (suicidal thoughts or wishes) on item 9 of the BDI-II, discontinuation will be initiated. Each rater has an emergency plan, and the respective participant will be shifted to inpatient or outpatient psychotherapy.

**Table 2:** Assessment tools utilized throughout the programme

Assessment timeframe	Assessment instrument
Pre (T1)	Self-assessment—questionnaires: BDI-II; QIDS-SR16; BAI; WHOQOL-BREF; SWOP-K9; WAI-SR; APOI; SASS; Bado
	Assessment by a therapist – Questionnaires: MINI; HRSD-24
During (T2)	Self-assessment—questionnaires: BDI-II; QIDS-SR16; BAI; WAI-SR; APOI
Post (T3)	Self-assessment—questionnaires: BDI-II; QIDS-SR16; BAI; WHOQOL-BREF; SWOP-K9; WAI-SR; APOI; SASS; Bado Assessment by a therapist—questionnaire: HRSD-24
Follow-up (T4)	Self-assessment—questionnaires: BDI-II; QIDS-SR16; WHOQOL-BREF; Bado Assessment by a therapist—questionnaire: HRSD-24

*Abbreviations*: *MINI*, Mini International Neuropsychiatric Interview; *BDI-II*, Beck Depression Inventory-II; *QIDS-SR16*, Quick Inventory of Depressive Symptomatology; *BAI*, Beck Anxiety Inventory; *WHOQOL-BREF*, WHO Quality of Life-BREF; *SWOP-K9*, Self-Efficacy, Optimism, and Pessimism Scale; *WAI-SR*, Working Alliance Inventory - Short Revised; *APOI*, Attitudes towards Psychological Online Interventions Questionnaire; *Bado*, basic documentation; *HRSD-24*, Hamilton Depression Scale; *SASS*, Social Activity Self-Assessment Scale

Subjects are allowed to attend ongoing psychotherapy. This reflects everyday treatment and ensures high external validity. Furthermore, it limits potential negative consequences due to the lack of treatment success, as additional treatment can be accessed, when needed. All potentially relevant factors for treatment outcome are taken into account for the statistical evaluation.

Voluntary termination was handled according to the study programme. Researchers will go beyond the protocol to contact dropped-out participants and try to continue the collection of data.

In case of harm induced by the trial, even though this is unlikely as depicted above, there is insurance coverage for the study participants provided by Selfapy’s public liability insurance GmbH. There are no guidelines for stopping the study. The study design was approved by the ethics committee of the medical faculty of the Charité University Medicine Berlin.

### Ethics and data protection

It is ensured that data can be not associated with personal data of the participant, e.g. name. In case of subsequent withdrawal from consent, the respective participant is identified via a code-word. Data is saved on CDs, which are stored in a safe and only accessible to a small number of people involved in the research project; data will be archived for 10 years after completion of the trial and then deleted.

### Assessments

Assessments will be made at four distinct points in time. Baseline (T1, study entrance), 6 weeks after baseline (T2), 3 months after baseline (T3, end of the programme) and 12 weeks after the completion of the programme (T4, follow-up). All other questionnaires are completed by the subjects (self-rating).

### Outcome measures

#### Primary outcome measure

The primary outcome measure is the change in the self-assessment of depressive symptoms after the completion of the programme using the BDI-II on all assessment points.

#### Secondary outcome measures

Changes in depressive symptoms will be additionally assessed at baseline (T1) and post (T3) after 12 weeks as well as at follow-up (T4) using observer-rated inventories HRSD-24 and the QIDS [25].

Furthermore, changes in the self-assessment of anxiety-related symptoms will be analysed using the BAI [26]. Changes in self-esteem will be evaluated using the SWOP-K9 [27]. Similarly, the SASS [28] questionnaire will be utilized to record the levels of social activation. The WHOQOL-BREF [29] will be used to assess the impact of the programme on physical changes and life satisfaction. The APOI captures attitudes towards online interventions [30].

The WAI [31] is an employment inventory for the collection of therapeutic alliances and is used to evaluate work alliances. The ACSA is a self-anchoring rating scale for subjective well-being that was originally developed as a simple method to measure the quality of life consecutively in the patient-physician relationship [32]. The Bado sheet is used to assess the impact of the programme on subjects’ use of the healthcare system, including contact with healthcare providers, the number of therapy sessions and the amount of contact with psychotherapists and psychiatrists.

### Engagement and usage measures for iCBT

The log files on the online “Selfapy” platform will be utilized to keep track of certain data. These data include but are not limited to the number of times a subject visits the platform, the amount of time elapsed between logins and the amount of correspondence shared between a subject and his/her respective therapist. As an addendum, the platform’s track and change functionalities enable the collection of data on the subject’s exact amount of usage on the platform. This includes the frequency of visits to the online modules, how much time a subject spends on each module and the modules that have been completed by the subject and in which order. All the aforementioned information will provide an overall picture of the efficiency and success of the individual elements of the programme.

### Sample size

The between-group effect size estimate is based on meta-analytic evidence for the effect size observed in unaccompanied psychological interventions (*d* = 0.28) [16]. Treatment effect sizes in a sample with mild to moderately depressed subjects may be quite low. We conservatively reduced the estimated effect size to *d* = 0.23 [33].

Based on this effect size, a power of 0.80 and an alpha level of 0.05, we need a total of 200 subjects to address our hypotheses. The sample size was further estimated based on an expected drop-out rate of 50%. Drop-out rates in previous studies that used similar interventions ranged between 9% [9, 10] and 50% [34]. Based on this assumption, we would need a total of 400 subjects. The sample size was calculated using G-Power [35]. In addition, a comparison will be drawn between the unaccompanied and the accompanied versions of the online programme. The previously expanded experimental groups are compared to a control group, resulting in a total of three study groups to which the subjects are randomly assigned in a ratio of 2:2:1 (accompanied group: *n* = 160, unaccompanied group: *n* = 160, control group: *n* = 80).

### Planned statistical analysis

The recorded data of the different measurement times (T1, T2, T3 and T4) for both intervention groups and the control group are analysed. The treatment results of all subjects (pre-post difference) are analysed using intention-to-treat (ITT) analyses.

The primary endpoint is the decrease in depressive symptoms in the BDI-II (Delta BDI-II) between study entrance (T1) and the end of the intervention (T3). A one-way ANOVA (within factor “group”) is performed to analyse the differences in the decrease of depressive symptoms between the intervention groups. A Bonferroni correction for multiple testing is applied to examine the expected group differences. This correction is carried out using the formula *α*/*n* (with alpha level *α* = 0.05 and *n* = number of tests) in order to lower the level of significance, make fewer results significant and thus the number of false-positive results reduce.

In an exploratory analysis, the basic effectiveness of the intervention compared to the control condition is examined (accompanied and unaccompanied group vs. control group) in order to counter the high drop-out rate.

In addition, the mean values including confidence intervals (95%) are recorded. The data are aggregated in the form of mean values and standard deviations. No interim analyses are planned.

To check the data obtained from the measuring instruments for normal distribution, the Kolmogorov-Smirnov test is calculated at the beginning. For this purpose, the mean values and standard deviations of each variable, as well as the Kolmogorov-Smirnov *Z*-value, are calculated, and the asymptotic significance (two-sided) is determined. Values below 0.05 indicate a violation of the normal distribution.

The intermediate measurements are analysed with regard to continuous outcome measures using repeated-measure ANOVAs. Independent *t* tests and *χ*^2^ tests are used to estimate the intergroup differences in sample characteristics prior to treatment.

Missing values in the data are replaced by multiple imputations, as well as “last-observation-carried-forward” (LOCF), “baseline-observation-carried-forward” (BOCF) and a “reference-based multiple imputation” method (“jump-to-reference” approach) to replace the missing values.

Effect size calculations to measure the therapeutic success (before and after the intervention) are carried out by dividing the difference between the respective group mean values by their pooled standard deviation.

Effect sizes under 0.2 are deemed small, and 0.5 moderate and 0.8 are deemed large [36].

All data evaluations are carried out without knowledge of the political group affiliation (blinded data evaluation). That means the evaluating person does not know which expression of the group variable indicates the representation to the IG and which the rights to the KG.

An external team was built, in order to monitor and recheck the execution of the planned statistical analysis.

## Discussion

The aim of this study is to evaluate the effectiveness of the online self-help programme “Selfapy” in a randomized-controlled-blinded parallel-group study. “Selfapy” was developed to become a low-threshold and affordable option for people dealing with depressive symptoms, especially for patients with subclinical to moderate depression symptoms (e.g. [16, 19]).

In this study, both accompanied and unaccompanied online intervention programmes are evaluated. This study will give further insight into the comparison of accompanied and unaccompanied variations within the same programme, which will add the value of the component of accompaniment (dismantling approach [37];).

In addition to our primary outcome (change of depressive symptoms), we will further investigate important other parameters such as life satisfaction, therapeutic relationships, social activation, self-esteem and attitudes. Additionally, an economic evaluation will be conducted from a healthcare perspective in future research.

Two weekly ACSA assessments in the intervention groups will allow us to identify meaningful patterns of early changes in life satisfaction during online treatment. These patterns will be investigated to predict the outcome at treatment termination and over the follow-up period, as well as drop-out rates or the number of times subjects participate in online treatment. These regular measurements may yield patterns that indicate the outcome of discontinuation of the treatment and allow for the follow-up period [38, 39].

A major strength of this study is the low-threshold criteria of inclusion, as anyone suffering from depressive symptoms can sign up through the study website without further requirements. These measures will improve the external validity of our study. This strength may also be regarded as a limitation, as it may result in a relatively heterogeneous group of patients [33]. The limitations of the study include the possibility of the parallel use of the health system, which could cause changes in symptoms, although we assess the healthcare use by questionnaires. It should also be taken into consideration that primary diagnoses other than depression are also included in the study.

The main article and several minor articles are pinned to the present study. The publication takes place in appropriate topic-specific journals. Any changes that differ from the present protocol will be openly communicated when the main article and any secondary articles are published.

## Conclusion

We evaluate the efficacy of the self-help programme “Selfapy” that offers low-threshold treatment to various patients also with mild to moderate depressive symptoms. By the dismantling approach, we can investigate the value of accompanied vs. unaccompanied variations within the same programme. Positive and meaningful results are expected in this study, which may have an impact on the acceptance and implementation of such programmes.

## Trial status

Regulatory approval was issued by the ethics committee of the Medical Faculty of the Charité University Medicine Berlin on 24 May 2019 (protocol version number is 2:00 dated 27 January 2021). Recruitment started on 24 June 2019; the number of recruited participants on 28 May 2020 was *n* = 298, and recruitment is ongoing.

## Trial registration

The trial has been registered on the German Clinical Trials Register (DKRS) (14 June 2019), with identifier number DRKS00017191.

The study protocol has been reported in accordance with the Standard Protocol Items: Recommendations for Clinical Interventional Trials (SPIRIT) guidelines.

## Data Availability

There are no plans to give public access to the participant level code. Only the study director, the study coordinator, the doctoral candidate and the student employees of the research group have access to the test data set.

## Abbreviations

ACSA: Anamnestic Comparative Self-Assessment
APOI: Attitudes towards Psychological Online Interventions Questionnaire
Bado: Basic documentation
BAI: Beck Anxiety Inventory
BDI-II: Beck Depression Inventory-II
CBT: Cognitive behaviour therapy
HRSD-24: Hamilton Depression Scale
CI: Confidence interval
iCBT: Internet-delivered cognitive behaviour therapy
MINI: Mini International Neuropsychiatric Interview
RCT: Randomized controlled trial
SASS: Social Activity Self-Assessment Scale
SMD: Standardized mean difference
SWOP-K9: Self-Efficacy, Optimism and Pessimism Scale
QIDS-SR: Quick Inventory of Depressive Symptomatology Self-Report
WAI: Working Alliance Inventory
WHOQOL-BREF: WHO Quality of Life-BREF
